# Bias against biologics in bariatric surgery: is it justified?

**DOI:** 10.1007/s00464-025-12046-z

**Published:** 2025-08-14

**Authors:** Jerome C. Anyalebechi, Brian Howard, Victoria Lyo, Mohamed Ali, Shushmita M. Ahmed

**Affiliations:** 1 Department of Surgery, University of California, Davis, Sacramento, CA USA; 2 School of Medicine, University of California, Davis, Sacramento, CA USA; 3 Center for Metabolic and Alimentary Science, University of California, Davis, Sacramento, CA USA; 4 Department of Surgery, University of California, Davis, Sacramento, CA USA

**Keywords:** Biological agents, Immunosuppression, Metabolic surgery, Autoimmune disorders

## Abstract

**Background:**

Obesity is a known risk factor for autoimmune disorders. Chronic steroid treatment among these patients poses greater surgical risk, but biological/immunomodulating agents (BIA) have allowed for safer alternatives. The effects of BIA monotherapy on postoperative complications following metabolic/bariatric surgery (MBS) have not been studied. We aimed to evaluate the effects of preoperative BIA use on post-metabolic/bariatric surgery outcomes.

**Methods:**

We conducted a retrospective matched-pair cohort study of patients who underwent bariatric surgery between 2012 and 2024. Patients were stratified into two groups: those with autoimmune disorders on BIA monotherapy vs matched controls. Patients were matched by age, BMI, and surgery type (Roux-en-Y gastric bypass [RYGB] vs vertical sleeve gastrectomy [VSG]). Paired T-tests were used to compare continuous variables. McNemar’s test was used to compare dichotomous variables.

**Results:**

Thirty patients (15 per group) were included in the study. Sixty percent of patients underwent VSG. Among biologic monotherapy patients, 46.7% had rheumatoid arthritis, and 66.7% of BIA were TNF inhibitors. BIAs were used within 12 months of surgery, stopped 6 weeks prior to surgery, and resumed 6 weeks after surgery. Although there was no difference in preoperative comorbidities, BIA group had a significantly higher ASA status (100% with ASA 3) compared to controls (60.0% with ASA 3, *p* = 0.03). Postoperatively, there were no differences in wound complications (6.7% BIA vs. 0% control, *p* = 1.00) or readmissions (0% BIA vs 6.7% control, *p* = 1.00). There were no anastomotic leaks, VTE, or death in either group. Six-month postoperative weight loss was equal between groups: (50.7 ± 15.4%EWL BIA vs 53.4 $\pm$ 19.8%EWL control, *p* = 0.89; %TWL (19.7 ± 6.1 [15.1–23.8] vs. 21.2 ± 5.9 [16.9–27.6], *p* = 0.78).

**Conclusion:**

Despite higher ASA status, patients on BIA did not have increased postoperative complications, and had similar weight loss to control patients after MBS. Our data suggests that when managed appropriately, patients on BIA have similar benefits from MBS without added risk.

While rates of obesity continue to increase worldwide, metabolic/bariatric surgery (MBS) remains the most effective and durable treatment for weight loss and obesity-related comorbidities [1–3]. With low rates of complications and high overall safety profiles, decisions to exclude patients from bariatric surgery are often surgeon driven and based on a patient’s anatomy, ability to tolerate surgery, and prohibitive risk factors increasing likelihood of complications.

Autoimmune disorders have often been historically considered a relative contraindication to MBS, both due to the disease itself as well as the immunosuppressive medications used for treatment [4]. The relationship of autoimmune disorders with obesity and MBS is complex. Obesity itself is a chronic, inflammatory disease, is a risk factor for development and flare ups of autoimmune conditions [5–7]. Outcomes following MBS are mixed. While some evidence has shown improved prognosis and reduced flares among some patients with autoimmune disorders, other studies cite greater postoperative hospitalization and intervention for disease progression [8–11]. Additionally, though emerging literature shows equivalent weight loss among patients with autoimmune disorders undergoing MBS, rates of postoperative complication rates are variable [12, 13].

It is unclear if complications among patients with autoimmune disorders are due to immunosuppressive medications or the underlying disease, as this is not clearly delineated in the literature. Previously, the mainstay treatment for autoimmune disorders was steroids. Chronic steroid use is associated with the development of bone fractures, hypertension, and various metabolic issues such as weight gain and hyperglycemia [14, 15]. In patients undergoing abdominal surgeries (including MBS), chronic steroid use increases the risks of postoperative wound infections, renal insufficiency, and pneumonia [16, 17]. Development of newer biologics/immunomodulating agents (BIA) has allowed for safer alternative therapies. Despite their overall immunosuppressive effects, BIA are targeted medications that have not been shown to increase postoperative complications [18, 19]. While their effect on surgical outcomes has been studied extensively in Crohn’s and ulcerative colitis literature, the effects of BIA (without concurrent steroid use) on outcomes following MBS has not been reported. Given the growing use of BIA, evaluating the effect of this treatment in the bariatric population is crucial [20, 21]. Thus, we aimed to evaluate the effects of preoperative BIA on post-MBS outcomes.

## Methods

A retrospective review of a prospectively maintained MBS database was performed at a single academic institution following approval by the institutional review board. Patients ≥ 18 years undergoing primary laparoscopic Roux-en-Y gastric bypass (RYGB) or laparoscopic vertical sleeve gastrectomy (VSG) between 2012 and 2024 were screened to identify those with autoimmune disorders documented to be on biological/immunomodulating agent (BIA) monotherapy (i.e. without concomitant steroid use) within 12 months of surgery. BIAs were stopped 6 weeks prior to surgery and resumed 6 weeks after surgery. This was an institutional protocol developed by the practice in place at the time of the study. During each patient's preoperative visit, it was verified that the patient had not received a dose of their BIA within 6 weeks of the scheduled operation. A matched-pair cohort was created by matching for age, body mass index (BMI), and procedure type (RYGB vs. VSG). Baseline characteristics collected included age, sex, BMI, race, medical comorbidities (hypertension, diabetes, cardiac disease [CAD], obstructive sleep apnea [OSA]), American Society of Anesthesiologists Physical Status Classification (ASA status), and procedure type (RYGB vs VSG).

Primary outcome was the prevalence of major 30-day postoperative complications. This included any wound complication, anastomotic or staple line leak, dehydration requiring intravenous fluids, venous thromboembolism(VTE), urinary tract infection (UTI), 30-day readmission, and death. Additionally, standardized weight loss outcomes, including percent excess weight loss (%EWL), percent total weight loss (%TWL), and body mass index (BMI) were collected at 6 and 12 months postoperatively [22]. These are reported with 95% Confidence Intervals (CI). Missing weight data were excluded from final analysis.

Statistical analysis was conducted using SPSS Statistics 29.0 from IBM (Armonk, NY, USA). Paired T-tests were used to compare continuous variables. McNemar’s test was used to compare dichotomous variables.

## Results

A total of 16 patients with autoimmune disorders on BIA monotherapy undergoing primary MBS were identified. Fifteen patients were successfully matched and included in the matched-pair analysis. One patient could not be matched due to high BMI (BMI 72). A complete list of autoimmune disorders and BIA is summarized in Table 1. Autoimmune disorders included rheumatoid arthritis (46.7%), psoriasis (26.7%), ankylosing spondylitis (6.7%), and ulcerative colitis (6.7%). BIA use was documented within 9 months preoperatively. The majority of BIA used were tumor necrosis factor (TNF) inhibitors (66.7%), with adalimumab and etanercept (both 26.7%) being the most commonly used agents (Table 1**)**.

**Table 1:**
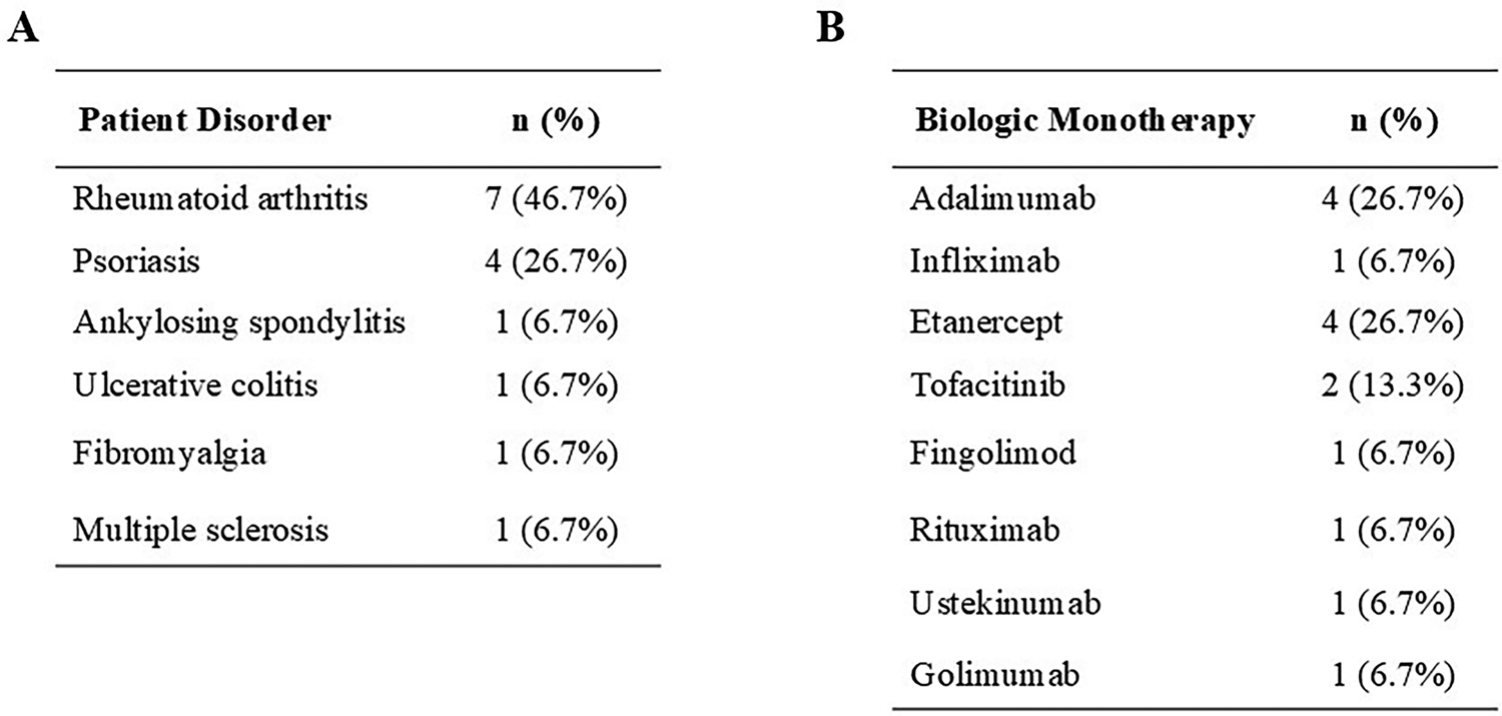
**A)** Types of autoimmune disorders identified. **B)** Types of biologic/immunomodulating agents used prior to and after bariatric surgery

There were no differences in the two groups in age (46.3 ± 11.2 years BIA vs. 47.4 ± 11.7 years control, *p* = 0.20), BMI (44.3 ± 9.1 kg/m^2^ BIA vs. 44.1 ± 8.2 kg/m^2^ control, *p* = 0.66), and surgery type (60.0% VSG in both groups). Similarly, there were no significant differences in gender or race between the groups. There were no differences in rates of preoperative obesity-related comorbidities, including: hypertension (66.7% BIA vs 60.0% control, *p* = 1.00), diabetes (33.3% BIA vs. 26.7% control, *p* = 1.00), obstructive sleep apnea (60.0% BIA vs. 66.7% control, *p* = 1.00), and cardiac disease (6.7% BIA vs. 20.0% control, *p* = 0.63). BIA monotherapy group had a statistically significant higher American Society of Anesthesiologist (ASA) status than control counterparts (ASA status 3: 100% in BIA group vs 60.0% in control group, *p* = 0.03) (Table 2).

**Table 2:**
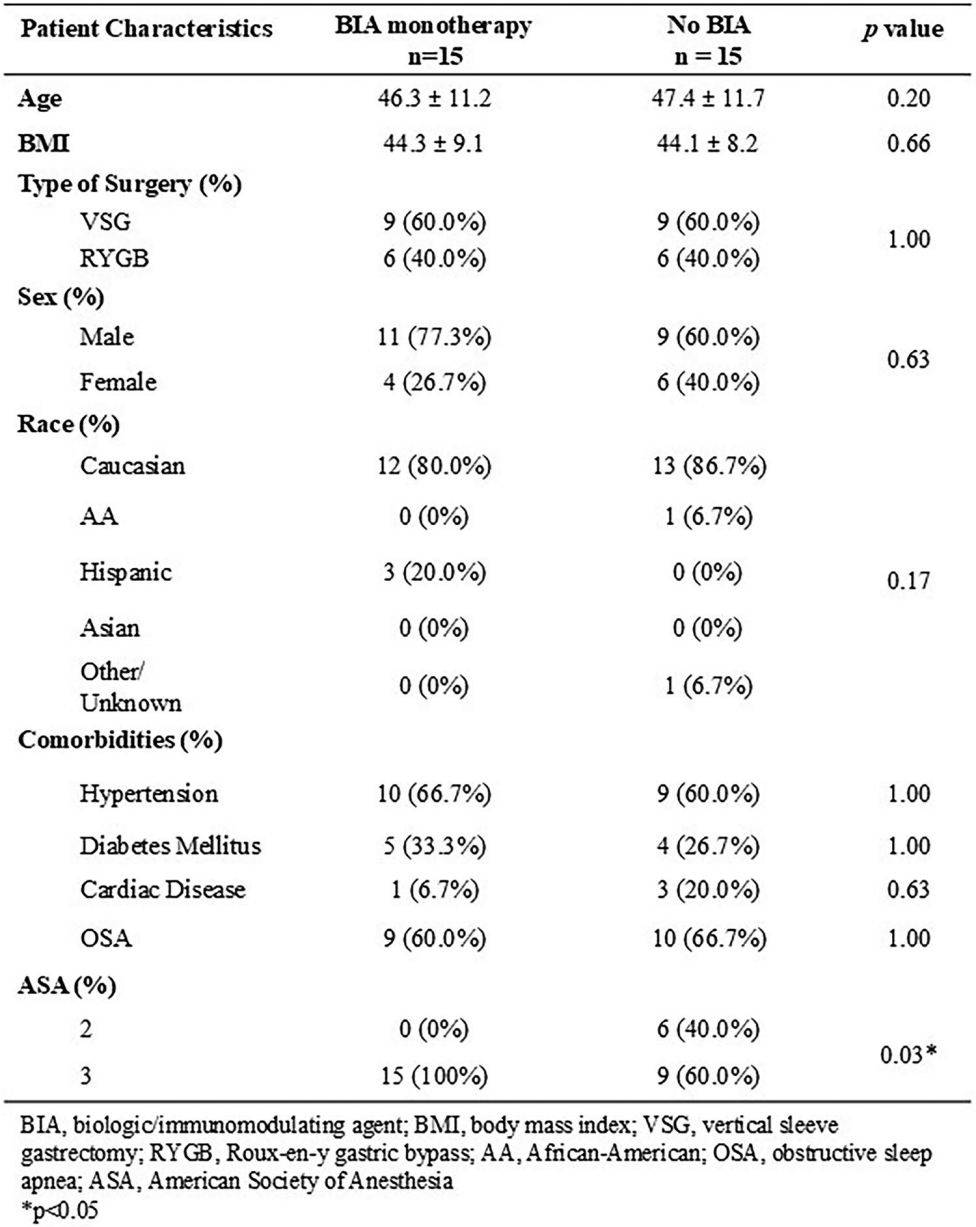
Baseline characteristics of patients on biologic/immunomodulating agents and their matched cohort

Postoperatively, there were no differences in 30-day complications between groups (Table 3). There was one wound complication (6.7%) in the BIA monotherapy group and none within the control group (*p* = 1.00). There were no readmissions among the BIA group, and one readmission in the control group (*p* = 1.00). Finally, there were no postoperative anastomotic leaks, VTE, UTI, or deaths in either group.

**Table 3:**
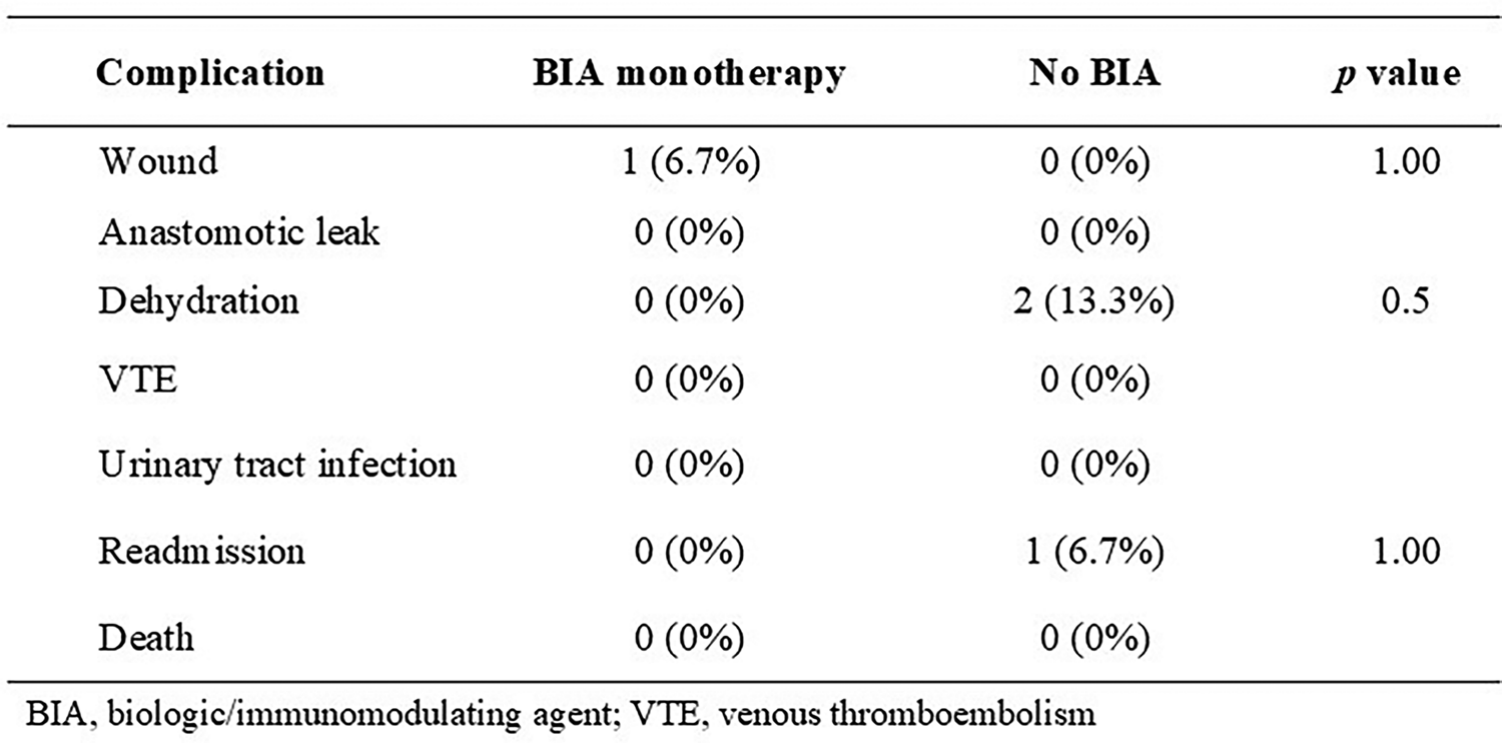
Complications within 30 days after bariatric surgery

**Fig. 1:**
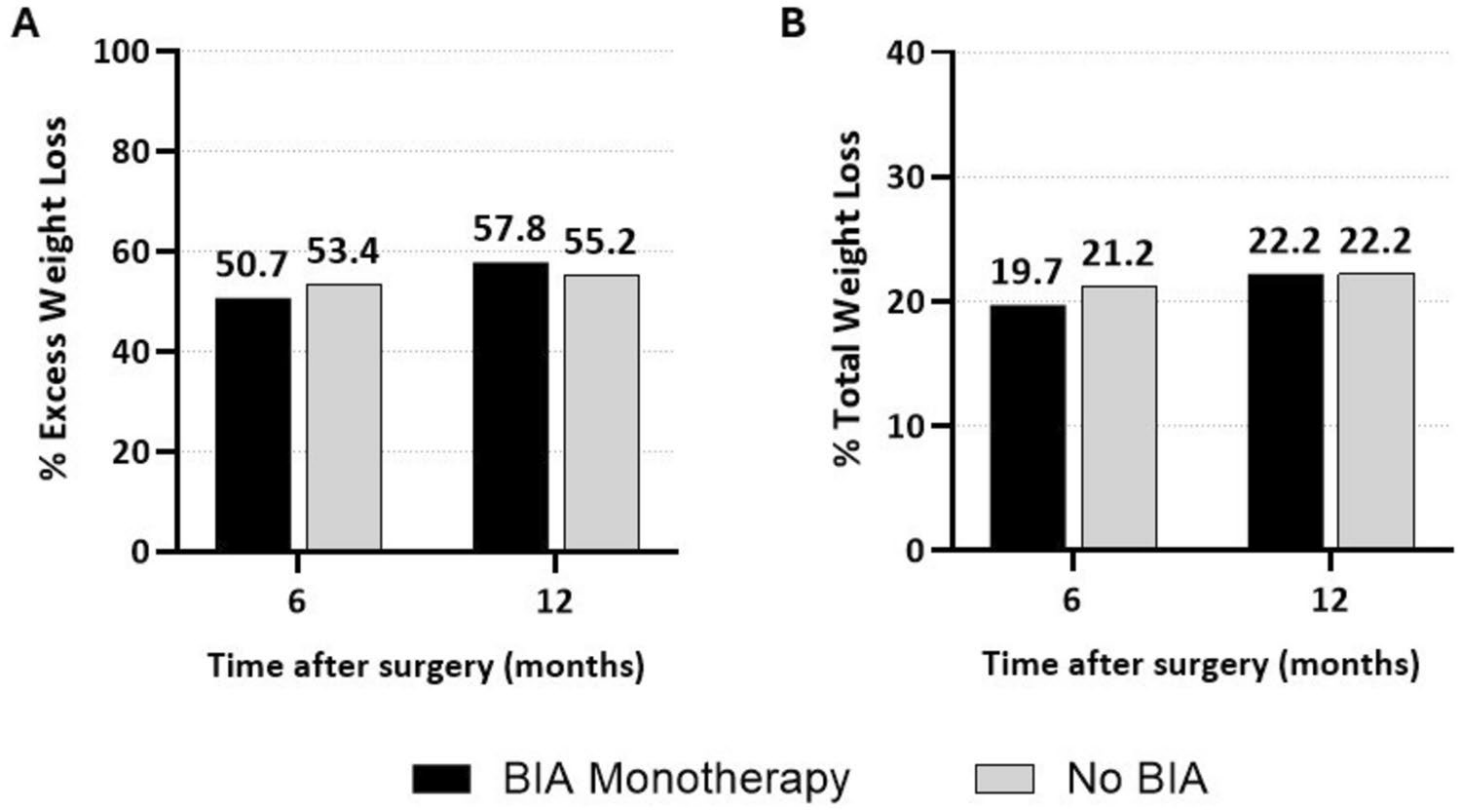
Weight loss outcomes after bariatric surgery. **A** Percent excess weight loss at 6- and 12-months. **B** Percent total weight loss at 6- and 12-months

Importantly, weight loss and BMI were similar between groups at all postoperative time points (Table 4). This includes outcomes at 6-months: %EWL (50.7 ± 15.4 [95% CI 43.7–65.7] vs. 53.4 ± 19.8 [35.0–72.8], *p* = 0.89), %TWL (19.7 ± 6.1 [15.1–23.8] vs. 21.2 ± 5.9 [16.9–27.6], *p* = 0.78), BMI (35.0 ± 7.0 [29.3–36.0] vs. 34.8 ± 7.5 [28.5–42.8], *p* = 0.71); and 12-months: %EWL (57.8 ± 14.3 [46.8–68.8] vs. 55.2 ± 27.4 [34.7–83.3], *p* = 0.88), %TWL (22.2 ± 11.3 [13.5–30.9] vs. 22.2 ± 9.1 [16.2–31.8], *p* = 0.90), and BMI (31.1 ± 2.7 [29.1–33.2] vs. 35.3 ± 8.9 [27.1–42.3], *p* = 0.67) (Fig. 1). Of note, 13 of 15 (86.7%) BIA patients and 11 of 15 (73.3%) control patients presented for 6-month follow-up while 9 of 15 (60.0%) BIA patients and 10 of 15 (66.7%) control patients presented for 12-month follow-up.

**Table 4:**
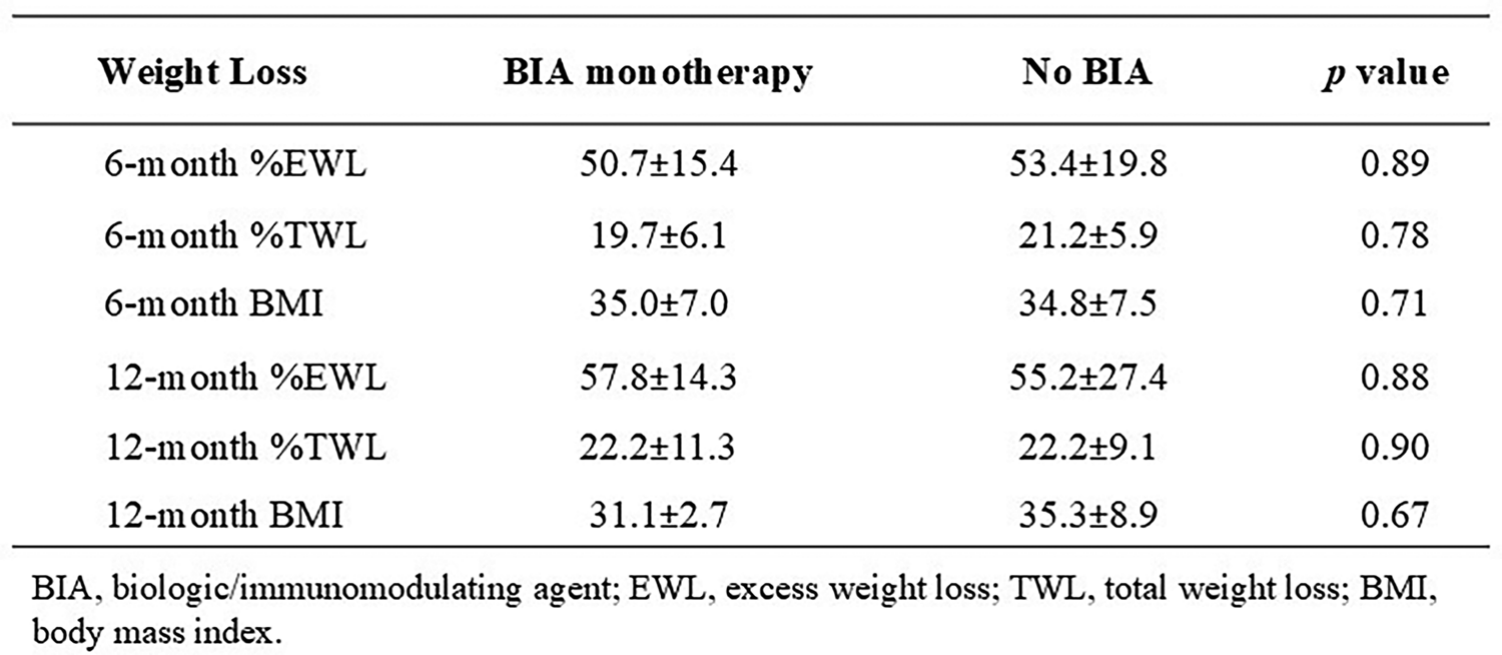
Weight loss outcomes at 6- and 12-months after bariatric surgery

## Discussion

As the prevalence of obesity and the use of biologic/immunosuppressive agents continue to rise, an increased intersection between BIA use and MBS is inevitable [23, 24]. The limited data on the effect of immunosuppressive medications on MBS outcomes are further confounded by the variability of agents included in these studies. For example, using the Metabolic and Bariatric Surgery accreditation and Quality Improvement Program (MBSAQIP) data registry, Hefler and colleagues showed that chronic immunosuppression was associated with increased risk of 30-day major complications (including wound infections and anastomotic leak) [12]. However, this study was limited by a retrospective database which a) did not specify how medications were managed perioperatively, and b) did not distinguish between patients on steroids vs alternative immunosuppressants. On the other hand, Maroun et al. found complication rates and weight loss among patients on immunosuppressive agents comparable to prior studies, but once again, a major study limitation was the inclusion of a large proportion of patients on chronic steroids (65%) [13].

Our study is unique in its inclusion of patients only on BIA monotherapy for autoimmune disease treatment. This is important in separating the known risks of chronic steroid use from that of BIA in the perioperative period. What is more, patients were matched to limit further confounders. Rates of hypertension, diabetes, OSA, and CAD among BIA patients were similar not only to controls, but to broader bariatric populations, as well [25, 26]. Despite this similarity, ASA status was higher among patients on BIA than in controls. This may be, in part, a reflection of the impact of autoimmune disease on the patients’ overall health. Nonetheless, BIA patients did not have increased complications in the postoperative period, and overall complication rates in both groups were consistent with prior studies [27]. This suggests that although patients on BIA are sicker, MBS may be as safe in this cohort as it is in the general population.

Weight loss is the primary determinant of MBS efficacy. Our study showed similar weight loss between both groups at 6 and 12-months postoperatively. What is more, with 60% of patients undergoing VSG, weight loss outcomes were expected and comparable to those reported among general bariatric patients and patients with IBD [4, 28]. This is especially important in light of evidence suggesting increased weight gain with biologic medications (e.g. TNF inhibitors). As the majority of our BIA patients were on TNF inhibitors, these findings are important in demonstrating effective weight loss despite BIA use.

As with any operation, appropriate patient selection in MBS is necessary to ensure a balance between safety and efficacy. In this study, we demonstrate that the presence of autoimmune disorders and use of biologic/immunomodulating agents alone are not reasons to exclude this population of patients from a life changing (and often lifesaving) intervention. This, however, is not without caveats. An important feature of our practice are the strict guidelines for perioperative immunosuppressive medication management. We work closely with patients’ BIA providers to pause these agents and ensure 6 weeks hiatus pre and postoperatively (12 weeks total). This may play an important role in the safety profile seen in our outcomes. Indeed, patients with obesity and autoimmune disorders on BIA are a vulnerable subset of the bariatric population. With appropriate evaluation and management, these patients stand to reap equal benefits from MBS without added risk.

## Limitations

Our study is limited by its retrospective nature and small sample size which may have been compounded by a possible attrition bias with 11 of 30 patients lost to follow up at 12 months. Currently, few patients on BIA undergo MBS. To offset these low numbers, patients were matched to controls by preoperative parameters. Propensity score matching can potentially provide more evenly matched populations by which to compare, but with a small sample size, case–control matching for a limited number of preoperative parameters limits the possibility of overmatching and having insufficient power to detect real differences between groups. Another potential limitation of the study is that those patients with autoimmune conditions who did undergo MBS do not represent the overall population of patients with autoimmune disorders and obesity, which may create a selection bias of patients who are lower risk for surgery. However, the selected patients’ higher ASA status indicates that these still represent a “sicker” patient population when compared to our matched population of patients with obesity without autoimmune conditions. Nonetheless, not including ASA as part of matching helps avoid weakening the statistical analysis of creating a bias *towards* finding no differences between the two study groups. An additional limitation of the study was the inability to report changes in disease patterns (i.e. remission or flares) during the perioperative period. This also includes any changes in dosing or type of BIA use outside of the reinitiation of therapy at 6 weeks postoperatively.

While our results are promising, they are not generalizable. As such, future studies with larger MBS cohorts of patients on BIA are needed to confirm our results. A multi-institutional study is one way to achieve this goal. Potentially of greater impact is through the national MBSAQIP database. Currently, the “immunosuppressive therapy” category groups steroid use and other immunosuppressive agents together. Dividing this into distinct steroid and BIA subcategories would allow a more nuanced analysis to better inform us of effects of steroids vs immunomodulators on MBS outcomes.

## Conclusion

To our knowledge, this is the first study evaluating the effect of biologic/immunomodulator agent monotherapy on MBS outcomes. When managed appropriately, biologic monotherapy in patients with autoimmune disorders undergoing MBS does not lead to increased postoperative complications or inferior weight loss. Therefore, the use of biologics alone should not exclude this population from undergoing a crucial treatment for obesity.
